# Comparative analysis of distinct genomic landscapes in young-onset g*BRCA1/2* breast cancer

**DOI:** 10.1172/jci.insight.203005

**Published:** 2026-04-28

**Authors:** Mwangala P. Akamandisa, Mingyi Xia, Wilson Cheah, Bradley Wubbenhorst, Kurt P. D’Andrea, Mengyao Fan, Jake S. Shilan, Dana Pueschl, Anupma Nayak, Hayley McKenzie, William Tapper, Ellen R. Copson, Ramsey I. Cutress, Susan M. Domchek, Diana M. Eccles, Katherine L. Nathanson

**Affiliations:** 1 Division of Translational Medicine and Human Genetics and; 2 Abramson Cancer Center, Perelman School of Medicine, University of Pennsylvania, Philadelphia, Pennsylvania, USA.; 3 University of Southampton, Southampton, United Kingdom.; 4 Department of Pathology and Laboratory Medicine,; 5 Division of Hematology and Oncology, and; 6 Basser Center for BRCA, Perelman School of Medicine, University of Pennsylvania, Philadelphia, Pennsylvania, USA.

**Keywords:** Genetics, Oncology, Breast cancer

## Abstract

Carriers of germline *BRCA1/2* pathogenic variants (g*BRCA1/2* PVs) have elevated young-onset breast cancer risk. To define the pretreatment genomic landscapes of young-onset g*BRCA*-associated breast cancer, we evaluated 136 treatment-naive tumors diagnosed before age 50 in the prospective POSH study and 66 noncarriers from The Cancer Genome Atlas. Using whole-exome sequencing, we analyzed somatic variation, allele-specific loss of heterozygosity (asLOH), homologous recombination deficiency (HRD), and single-base substitution (SBS) signatures. gBRCA1 and gBRCA2 breast cancers had high rates of asLOH but differed significantly in average HRD scores and median SBS composition of signatures SBS1 (aging-associated), SBS18 (ROS-associated), and SBS3 (HRD-associated). Compared with g*BRCA2* tumors, g*BRCA1* tumors with asLOH were significantly enriched for alterations in hallmark ROS, DNA repair, and epithelial-mesenchymal transition pathways. In ER-positive, HER2-negative tumors from g*BRCA1/2* carriers compared with noncarriers, we found significant enrichment of *RB1*, *TP53*, *FAT1*, and *MYC* single-nucleotide variants, indels, and copy number variants associated with CDK4/6 inhibitor (CDK4/6i) resistance. Together, these findings demonstrate significant differences between g*BRCA1*- and g*BRCA2*-associated breast cancers, and preexisting CDK4/6i resistance mechanisms, supporting prospective trials comparing individualized therapy for g*BRCA1* versus g*BRCA2* carriers and comparing poly(ADP-ribose) polymerase inhibitors versus CDK4/6i for ER-positive g*BRCA1/2*-associated breast cancer.

## Abstract 2

The largest genomic study of young women with *BRCA1* and *BRCA2*-associated breast cancer provides insights into the landscape of differences in these tumors.

## Introduction

Approximately 12% of young-onset breast cancer patients (first diagnosis at age ≤40 years) and 30% of very-young-onset breast cancer patients (first diagnosis at age ≤30 years) carry germline *BRCA1* or *BRCA2* pathogenic variants (g*BRCA1/2* PV) (1–4). g*BRCA1/2* PV carriers have an elevated lifetime breast cancer risk of 60%–80% (5–9). The Prospective Study of Outcomes in Sporadic versus Hereditary Breast Cancer (POSH) is a prospective cohort study investigating differences in outcomes between hereditary (g*BRCA1/2*-associated) and sporadic young-onset breast cancer (10). Enrollment of more than 2,700 women diagnosed with breast cancer at age ≤40 (<50 if a known g*BRCA1/2* PV carrier) was performed in the United Kingdom between 2000 and 2008. The study demonstrated a 5-year survival advantage but no survival difference by year 8 after diagnosis for women with estrogen receptor–positive (ER-positive) breast cancer compared with those with ER-negative disease and similar overall survival (OS) between young-onset female breast cancer patients with and without g*BRCA1/2* PVs (3, 11). Genomic features such as allele-specific loss of heterozygosity (asLOH) were not considered in the primary analysis.

*BRCA1* and *BRCA2* are critical for repairing double-stranded DNA breaks through homologous recombination, and complete loss of *BRCA1/2* function through asLOH is associated with homologous recombination deficiency (HRD) (12–14). Although both proteins function in the same pathway, some broad differences in somatic features between g*BRCA1* and g*BRCA2* tumors have been reported. g*BRCA1* tumors are mostly ER negative, whereas g*BRCA2* tumors are mostly ER positive (15, 16). Compared with g*BRCA2* tumors, g*BRCA1* tumors harbor high frequencies of *TP53* PVs and greater amounts and distinct patterns of copy number variants (CNVs) (17–22). Losses of chromosome 2q (chr2q), chr4p, chr4q, chr5q, and chr12q are frequent in g*BRCA1*-associated tumors, whereas losses of chr6q and chr13q occur in g*BRCA2*-associated tumors (21–23). Furthermore, g*BRCA1*-associated tumors exhibit greater activation of genes involved in DNA repair than g*BRCA2*-associated tumors (24). However, the full landscape of differences between g*BRCA1* and g*BRCA2* tumors remains incomplete, as previous studies have been limited by low numbers of carriers, with the largest having only 64 g*BRCA1/2* carriers, and by the use of older comparative genome hybridization technologies (17, 21). It is still unknown whether there are any molecular differences between g*BRCA1* and g*BRCA2*, other than ER status, that have potential therapeutic utility.

g*BRCA1/2*-associated tumors are sensitive to poly(ADP-ribose) polymerase inhibitors (PARPi) in both metastatic and high-risk early breast cancers (25–29). Adjuvant PARPi significantly improves OS in early-stage, high-risk disease (30) and improves progression-free survival in metastatic disease (27, 28). In ER-positive, HER2-negative tumors, adjuvant cyclin-dependent kinase 4/6 inhibitors (CDK4/6i) combined with endocrine therapy improve invasive disease–free survival in early-stage disease, and significantly increase OS in metastatic disease, compared with endocrine therapy alone (31–34). Thus, both PARPi and CDK4/6i confer clinically meaningful benefit in early and metastatic breast cancer.

Despite the efficacy of both PARPi and CDK4/6i, primary and secondary resistance to each therapy has been observed (28, 30, 35–40). PARPi resistance most commonly arises through reversion mutations of g*BRCA1/2* (41). Absence of asLOH (37) also may confer primary resistance to PARPi in g*BRCA1/2* PV carriers (14, 42). We previously reported differences in asLOH frequency between g*BRCA1* and g*BRCA2* breast cancers from PV carriers unselected for age (90% vs. 54%); in the current study, all patients were under age 50 (92.6% diagnosed ≤40) (14). CDK4/6i impede cell proliferation by inhibiting the phosphorylation of the retinoblastoma protein (Rb) during the cell cycle (43, 44). Genomic alterations, such as *RB1* loss and *MYC* amplification, have been implicated in resistance to CDK4/6i (40, 45).

In young-onset pretreatment g*BRCA1/2*-associated breast cancer, the frequency of asLOH and its impact on survival remain unclear, as does the landscape of genomic alterations associated with response to CDK4/6i. An improved understanding of the distinct genomic landscapes of treatment-naive g*BRCA1* and g*BRCA2* PV–associated breast cancer may inform current treatment selection, particularly in ER-positive, HER2-negative tumors for which both PARPi and CDK4/6i are treatment options, and may inform strategies for possible future therapy development. Thus, we performed comprehensive genomic profiling of treatment-naive, primary non-metastatic breast tumors using whole-exome sequencing (WES) of matched tumor-germline DNA in 136 breast cancer patients with g*BRCA1/2* PVs from the POSH cohort.

## Results

### Cohort description.

**Figure 1:**
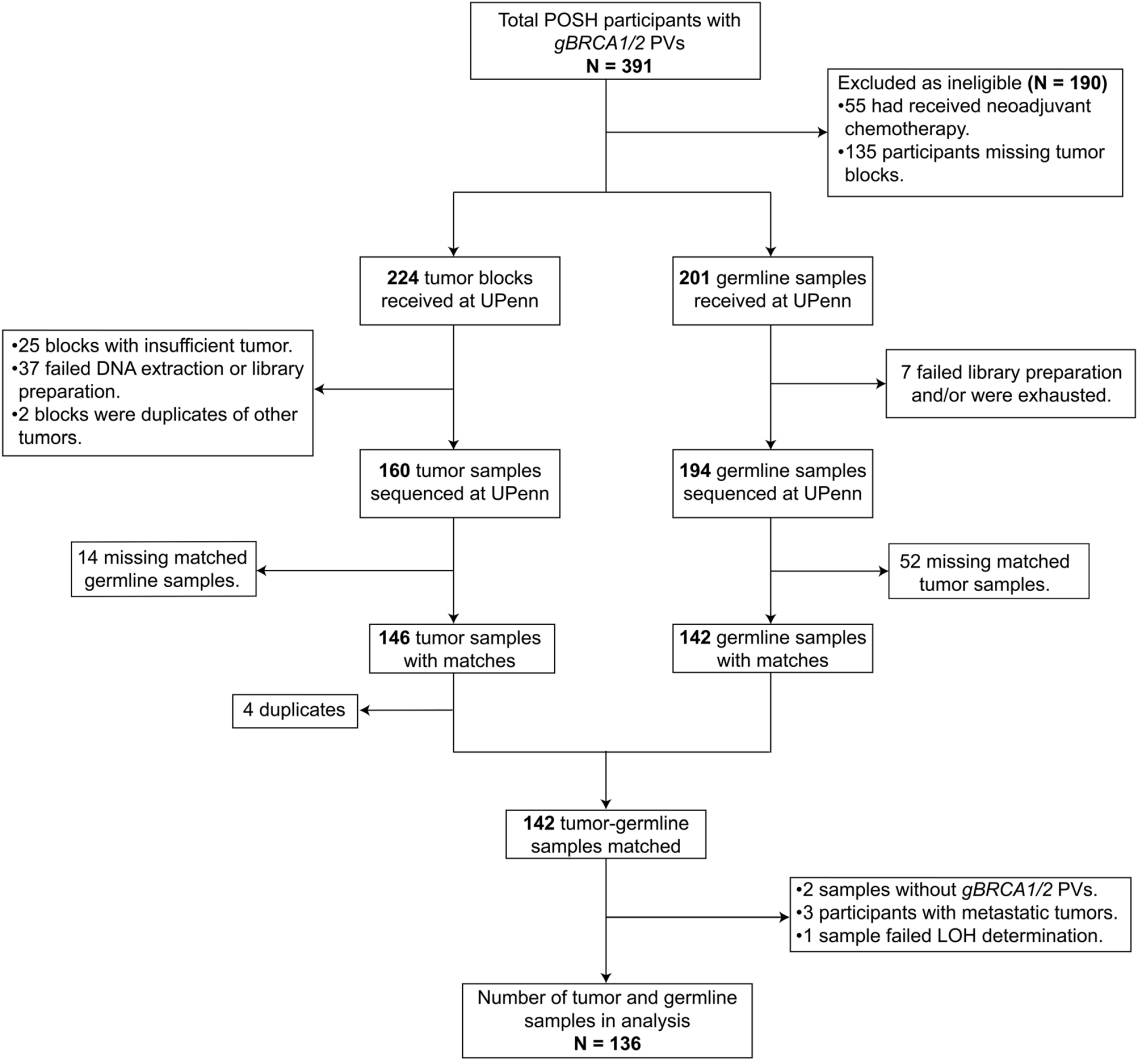
CONSORT diagram of POSH study samples.

We evaluated WES results of 136 treatment-naive matched tumor-germline samples from g*BRCA1/2*-positive women diagnosed with young-onset breast cancer participating in the POSH study (Figure 1, Supplemental Figure 1, and Supplemental Tables 1 and 2; supplemental material available online with this article; https://doi.org/10.1172/jci.insight.203005DS1). Eighty-six (63.2%) and 50 (36.8%) women had g*BRCA1* and g*BRCA2* germline PVs, respectively. The median age at diagnosis was 36 years, and 92.6% of women were age ≤40 (Table 1). Overall, 65 women (47.8%) had ER-positive tumors. Demographic, tumor pathology, and treatment data are shown in Table 1.

### Molecular features in breast cancers.

**Figure 2:**
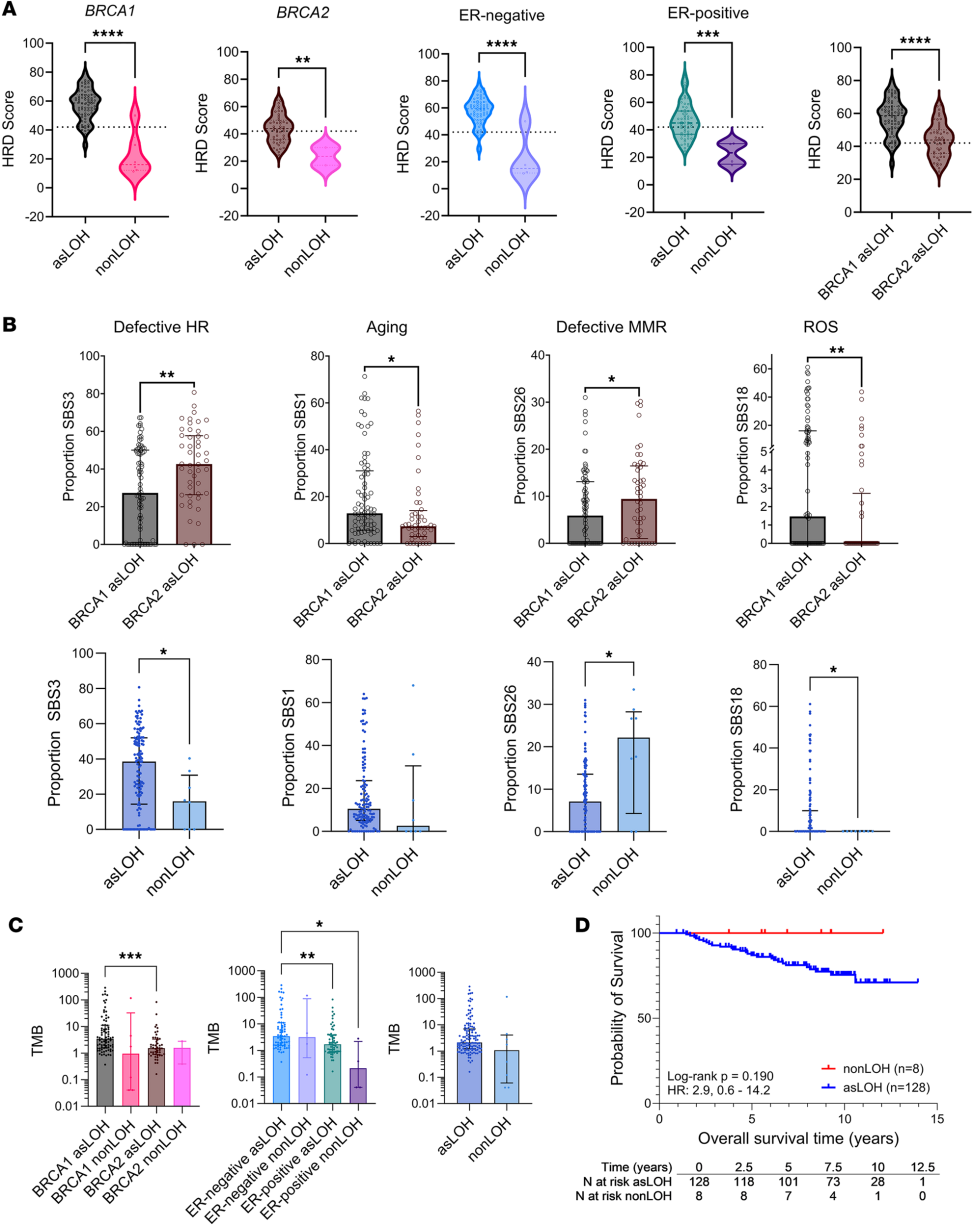
Molecular features of breast cancer in germline *BRCA1* and *BRCA2* pathogenic variant carriers with overall survival. (**A**) Homologous recombination deficiency (HRD) scores in tumors with allele-specific loss of heterozygosity (asLOH) compared with those without, stratified by germline variant gene and ER status using 2-sided *t* tests. Dotted black line is HRD score = 42. (**B**) Median of the proportion of single-base substitution signature 3 (SBS3), SBS1, SBS26, and SBS18 in tumors with asLOH by gene and in tumors with asLOH and those without, compared using Mann-Whitney non-parametric tests. Error bars are interquartile range (IQR). HR, homologous recombination; MMR, DNA mismatch repair. (**C**) Median tumor mutational burden (TMB) by asLOH status, germline gene variant, and ER status, compared using Kruskal-Wallis and Mann-Whitney non-parametric tests. Error bars are IQR. (**D**) Overall survival by asLOH status, evaluated using the Kaplan-Meier estimator and log-rank test. *P < 0.05, **P < 0.01, ***P < 0.001, ****P <.0001.

We evaluated the genomic landscapes of the breast cancers, including asLOH, HRD, tumor mutational burden (TMB), and single-base substitution (SBS). Tumors had high rates of asLOH when stratified by gene (93% for g*BRCA1* and 96% for g*BRCA2*) and by ER status (94.4% for ER negative and 93.8% for ER positive) (Supplemental Table 3). HRD scores were significantly higher in tumors with asLOH compared with tumors without asLOH (nonLOH) in both g*BRCA1* (57.4 ± 1.3 vs. 22.6 ± 6.1, *P* < 0.0001) and g*BRCA2* (43.7 ± 1.5 vs. 23.5 ± 6.5, *P* = 0.005) PV carriers and when grouped by ER status (ER-negative tumors: 57.6 ± 1.3 vs. 22.8 ± 9.2, *P* < 0.0001; ER-positive tumors: 46.4 ± 1.6 vs. 22.8 ± 4.1, *P* < 0.001) (Figure 2A and Supplemental Table 3). g*BRCA1* tumors with asLOH had significantly higher HRD scores than g*BRCA2* tumors with asLOH (57.4 ± 1.3 vs. 43.7 ± 1.5, *P* < 0.0001) (Figure 2A).

SBS3, the signature associated with g*BRCA1/2* PVs (37), was the most abundant signature in all tumors with an average contribution of 32.8% (Supplemental Figure 2). SBS1 (aging-associated), SBS26 (mismatch repair deficiency–associated), and SBS18 (reactive oxygen species–associated) were the next most abundant signatures (37). g*BRCA1* tumors had significantly higher median percentage proportional contributions of SBS1 (12.9 vs. 7.3, *P* = 0.013) and SBS18 (1.4 vs. 0, *P* = 0.007), and significantly lower proportions of SBS3 (27.3 vs. 42.6, *P* = 0.002) and SBS26 (5.9 vs. 9.4, *P* = 0.049), than g*BRCA2* tumors. The median percentage proportional contributions of SBS3 and SBS18 were significantly higher in tumors with asLOH than in nonLOH tumors (SBS3: 38.5 vs. 15.9, *P* = 0.023; SBS18: 0 vs 0, *P* = 0.023) (Figure 2B and Supplemental Table 4).

Tumors with g*BRCA1* asLOH had higher median TMB than those with g*BRCA2* asLOH (3.4, IQR 1.6–11.2, vs. 1.5, IQR 0.9–3.4, *P* = 0.001). ER-positive tumors with asLOH had significantly lower median TMB than ER-negative tumors with asLOH (1.7, IQR 0.9–3.9, vs. 3.4, IQR 1.6–11.0, *P* = 0.008) (Figure 2C). However, there was no difference in median TMB between tumors with and without asLOH. Tumors with TMB ≥ 10 have been found to be responsive to treatment with the anti–PD-1 monoclonal antibody pembrolizumab (46, 47). In our cohort, slightly more tumors with asLOH (24 of 128 [18.8%]) had TMB ≥ 10 than those without asLOH (1 of 8 [12%]), but it was not notable.

We evaluated breast cancer OS by tumor molecular features. Survival in women with nonLOH tumors was 100% throughout the 8.2-year median follow-up period but not statistically significantly different from that of women with tumors with asLOH (Figure 2D). Favorable survival for women with nonLOH tumors remained when the analysis was stratified by germline variant gene or by ER status (Supplemental Figure 3). The clinical characteristics of the nonLOH tumors are shown in Supplemental Table 5. Although not significantly so, women with HRD-high (≥42) tumors and those with SBS26 tended to do worse (Supplemental Figure 4, A and B). Women with tumors with SBS3 had significantly lower OS than those without (HR 4.46, *P* = 0.033). Similarly, when stratified by gene and ER status, women with SBS3 tumors had a decreased OS, albeit not to a significant degree (Supplemental Figure 4, C–G).

### Copy number variation in tumors.

**Figure 3:**
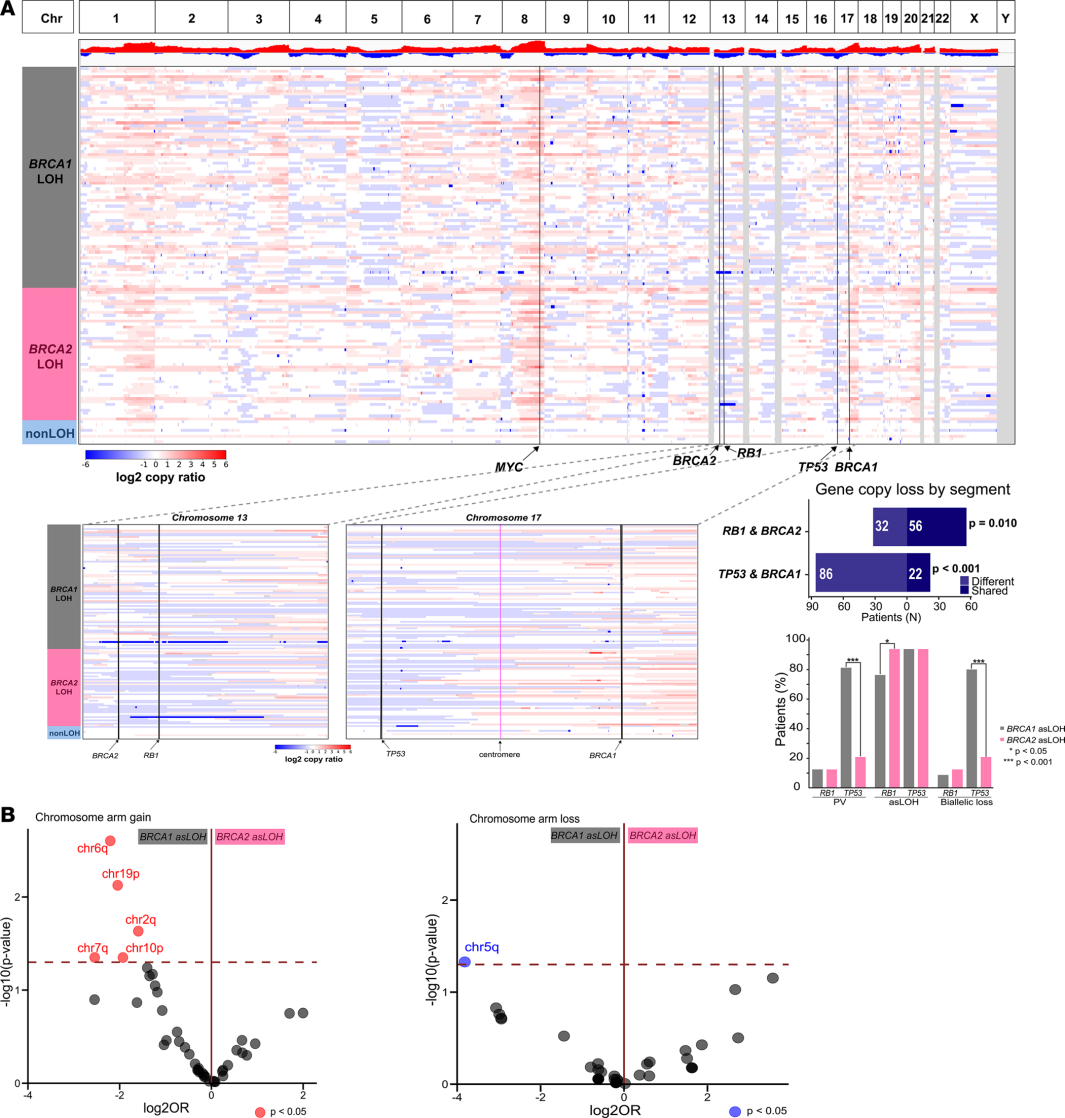
Copy number variation in tumor samples. (**A**) Landscape of copy number variants in tumors. Inset shows zoomed-in images of chromosomes 13 and 17 and the quantified values of times that *RB1* shares a copy number loss segment with *BRCA2* and *TP53* shares one with *BRCA1*, evaluated using a χ^2^ test. Vertical bar graph shows the frequency of *RB1* and *TP53* pathogenic variants (PVs), asLOH, and biallelic loss in *BRCA1* and *BRCA2* tumors with asLOH, evaluated using Firth logistic regression. *P < 0.05, ***P < 0.001. (**B**) Enrichment of chromosome arm gains and losses in tumors with *BRCA1* asLOH compared with those with *BRCA2* asLOH, adjusted for ER status. Enrichment was performed using Firth logistic regression. Dashed line is –log_10_(*P* value) = 1.3.

WES enabled copy number analysis, which revealed chromosome arm chr1q and chr8q gains, both prevalent in breast cancers (48), in all tumors (Figure 3A). Eighty-eight and 108 tumors had single-copy loss of *RB1* and *TP53*, respectively. Copy loss of *RB1* and *BRCA2*, both located on chromosome 13, occurred on the same segment significantly more frequently than expected by chance (56/88, *P* = 0.010), whereas copy loss of *TP53* and *BRCA1*, both on chromosome 17, occurred on the same segment significantly less frequently than expected by chance (22/108, *P* < 0.001) (Figure 3A). Thus, it appears that *RB1* and *BRCA2* loss are non-independent, whereas copy number loss of *TP53* and *BRCA1* are independent of each other. The loss of *RB1* (*RB1* asLOH) occurred significantly more frequently in g*BRCA2* asLOH tumors than in g*BRCA1* asLOH tumors; however, there was no difference in the frequency of *RB1* PVs or *RB1* biallelic loss. Conversely, *TP53* asLOH was not different between g*BRCA1* asLOH and g*BRCA2* asLOH tumors, although g*BRCA1* asLOH tumors had significantly more *TP53* PVs and *TP53* biallelic loss than g*BRCA2* asLOH tumors (Figure 3A).

We observed differences in chromosome arm copy number between tumors with g*BRCA1* asLOH and g*BRCA2* asLOH (Supplemental Figure 5). After adjustment for ER status (49), chr6q, chr19p, chr2q, chr7q, and chr10p gains and chr5q copy number were enriched significantly in tumors with g*BRCA1* asLOH compared with those with g*BRCA2* asLOH (Figure 3B).

### Enrichment of gene-level alterations.

To further define the complete genomic landscape of the tumors, we assessed gene-level somatic alterations (pathogenic single-nucleotide variants/indels and CNVs) in 598 cancer-associated genes (Supplemental Table 2). We found 4,146 pathogenic single-nucleotide variants (SNVs)/indels and 4,205 pathogenic CNVs in 572/598 genes (Supplemental Table 6). Pathogenic SNVs/indels in the 50 most commonly altered genes are shown in Supplemental Figure 6.

**Figure 4:**
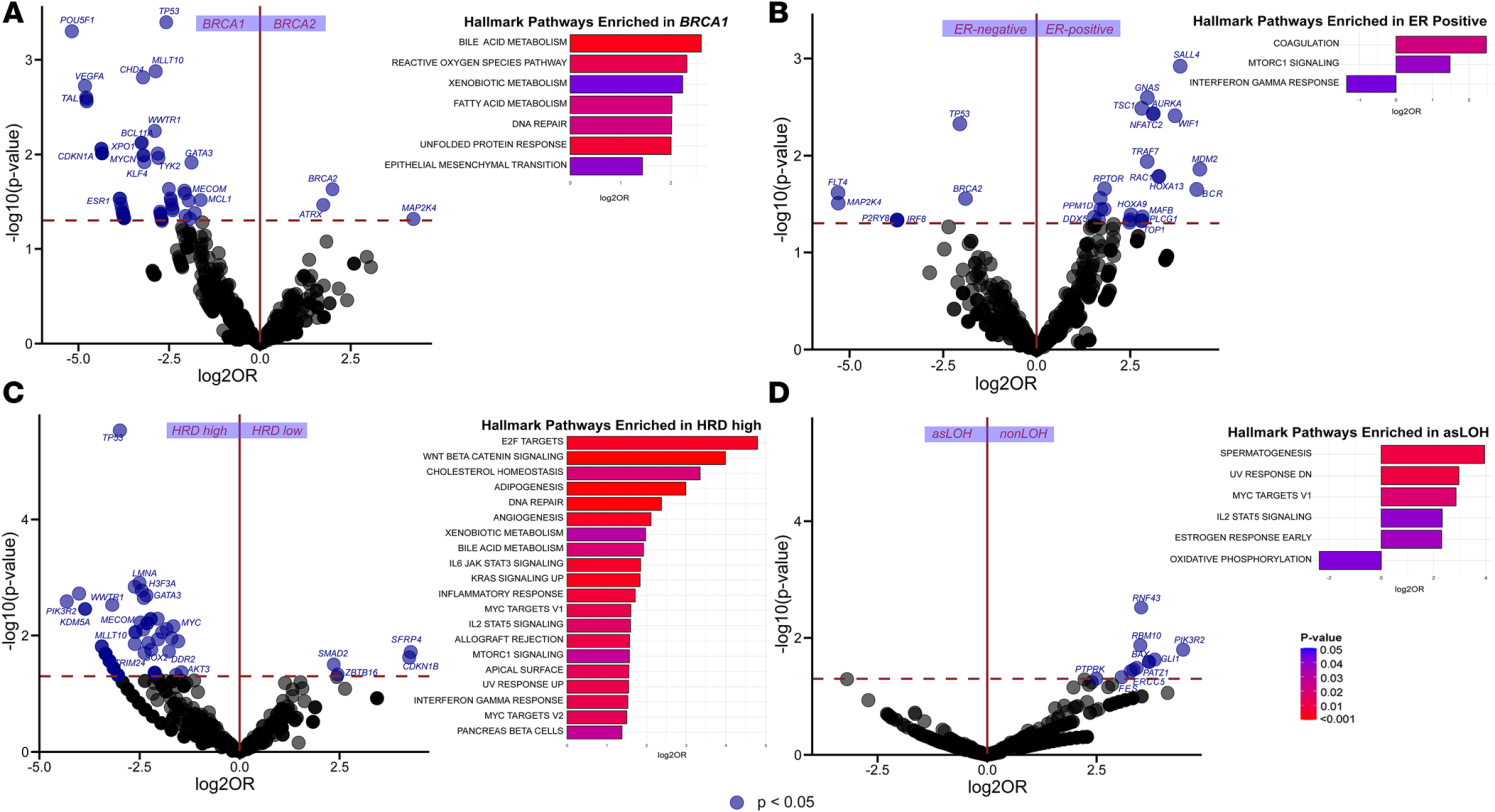
Differential enrichment of somatic variants, inclusive of pathogenic single-nucleotide variants, indels, and copy number alterations, and hallmark pathway enrichment in breast cancers. Enrichment of somatic variants and enrichment of Hallmark Pathway alterations by germline variant gene adjusted for ER status (**A**); by ER status adjusted for germline variant gene (**B**); by HRD (high ≥42) adjusted for asLOH (**C**); and by asLOH stratified by germline gene variant (**D**). Enrichment analysis was performed using Firth logistic regression. Dashed line is –log_10_(*P* value) = 1.3.

When we compared pathogenic SNVs/indels and CNVs in tumors from g*BRCA1* PV carriers with those from g*BRCA2* PV carriers, with adjustment for ER status, we observed a larger number of somatic alterations significantly enriched in g*BRCA1* tumors (Figure 4A and Supplemental Table 7). Pathway analysis revealed enrichment of pathogenic SNVs/indels/CNVs in genes in multiple hallmark pathways (50), including the reactive oxygen species (ROS) pathway, DNA repair, and epithelial-mesenchymal transition, in g*BRCA1* tumors compared with g*BRCA2* tumors (Figure 4A).

With adjustment for g*BRCA1/2* PV carrier status, ER-positive tumors had greater enrichment of pathogenic somatic SNVs/indels/CNVs compared with ER-negative tumors (Figure 4B and Supplemental Table 8) and were enriched for alterations in genes in the coagulation and mTORC1 signaling hallmark pathways. ER-negative tumors were enriched for alterations in the interferon-γ response pathway (Figure 4B). The total number of genes with SNVs/indels/CNVs and with SNVs/indels was higher in ER-negative tumors compared with ER-positive tumors when the analysis was performed without adjustment for g*BRCA1/2* PV status, as seen in previous studies (Supplemental Figure 7) (51).

More genes were enriched in SNVs/indels and CNVs in HRD-high tumors compared with HRD-low tumors, adjusted for LOH status (Figure 4C and Supplemental Table 9). Genes in the E2F targets and WNT/β-catenin signaling pathways were the most highly enriched in HRD-high tumors (Figure 4C). Tumors without asLOH were enriched for alterations in the oxidative phosphorylation pathway compared with those with asLOH (Figure 4D and Supplemental Table 10).

### Presence of CDK4/6 resistance alterations.

**Figure 5:**
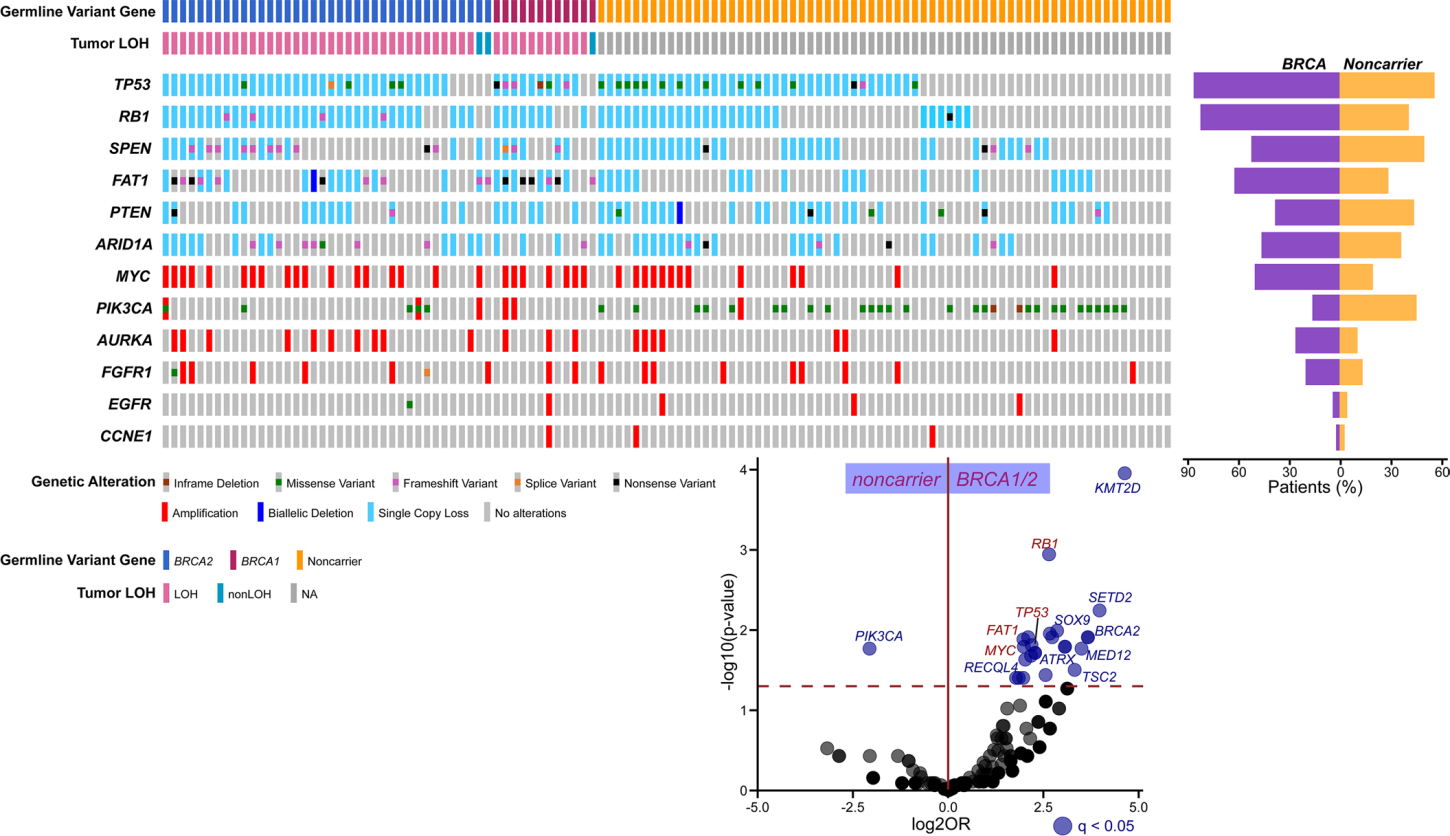
Enrichment of somatic variants associated with resistance to CDK4/6 inhibitors in ER-positive, HER2-negative tumors from germline *BRCA1/2* carriers in POSH compared with noncarriers from TCGA. Oncoprint of somatic variants in tumors, frequency of somatic variants, and enrichment of variants in POSH versus TCGA samples. Enrichment analysis was performed using Firth logistic regression. Dashed line is –log_10_(*P* value) = 1.3; *q* is the false discovery rate–adjusted *P* value.

Genetic alterations implicated in acquired and intrinsic resistance include alterations in *RB1*, *TP53*, *AURKA*, *MYC*, *CCNE1*, *SPEN*, *FAT1*, *ARID1A*, *PTEN*, *FGFR1*, and *EGFR* (40, 45, 52–58). We evaluated the presence of somatic alterations (SNVs/indels; and CNVs, including single-copy loss) implicated in resistance to CDK4/6i in ER-positive, HER2-negative tumors within the POSH cohort, and we found that all tumors contained at least one alteration implicated in resistance (Figure 5) (54). Genes implicated in CDK4/6i resistance exhibited various patterns of alteration in g*BRCA1* and g*BRCA2* PV carriers in ER-positive, HER2-negative tumors from the POSH cohort (Figure 5). Alterations in *RB1*, primarily consisting of single-copy loss (asLOH), were more frequent in g*BRCA2* tumors (33/38, 86.8%) than in g*BRCA1* tumors (8/12, 66.7%). Additionally, 4 tumors in g*BRCA2* PV carriers had frameshift PVs in *RB1*. PVs in *TP53* were more frequent in g*BRCA1* tumors (6/12, 50%) than in g*BRCA2* tumors (5/38, 13%), although the overall prevalence of *TP53* alterations was similarly high in both groups (83.3% in g*BRCA1* tumors and 86.8% in g*BRCA2* tumors) when single-copy-number loss was considered. Other CDK4/6i resistance–associated alterations observed in g*BRCA1* and g*BRCA2* tumors were in *SPEN* (g*BRCA1*: 50%; g*BRCA2*: 52.6%), *AURKA* (g*BRCA1*: 25%; g*BRCA2*: 26.3%), *ARID1A* (g*BRCA1*: 33.3%; g*BRCA2*: 50.0%), *FAT1* (g*BRCA1*: 83.3%; g*BRCA2*: 55.3%), *PTEN* (g*BRCA1*: 50.0%; g*BRCA2*: 34.2%), *MYC* (g*BRCA1*: 58.3%; g*BRCA2*: 47.4%), *EGFR* (g*BRCA1*: 8.3%; g*BRCA2*: 2.6%), and *CCNE1* (g*BRCA1*: 8.3%; g*BRCA2*: 0%).

To determine whether g*BRCA1/2* carriers were more likely than noncarriers to have somatic alterations in CDK4/6i resistance–associated genes, we compared rates of alteration between carriers from POSH and noncarriers from The Cancer Genome Atlas (TCGA) (Supplemental Figure 8), all with ER-positive, HER2-negative tumors. To mitigate any potential bias in sequencing coverage across the 2 cohorts, we limited our analysis to genes with at least 1 alteration detected in the TCGA cohort (Supplemental Table 8). We found statistically significant enrichment of alterations in several genes in tumors from the POSH cohort compared with those from TCGA, including *RB1* (OR 6.3, 95% CI 2.8–15.4, adjusted *P* value [*P*_adj_] = 0.001), *TP53* (OR 4.6, 95% CI 1.9–12.1, *P*_adj_ = 0.017), *FAT1* (OR 3.9, 95% CI 1.84–8.7, *P*_adj_ = 0.013), and *MYC* (OR 4.0, 95% CI 1.8–9.1, *P*_adj_ = 0.017), all of which are associated with resistance to CDK4/6i (Figure 5).

## Discussion

We performed WES on treatment-naive matched tumor germlines from 136 young g*BRCA1/2* PV carriers from the POSH cohort and found high levels of asLOH, over 90%, in all tumors regardless of germline variant gene and tumor ER status. No deaths were seen in women with a nonLOH breast cancer over the 8.2-year follow-up period; however, these numbers were small, and survival differences with asLOH tumors were not statistically significant. Presence of SBS3 was associated with a significantly lower OS, though numbers were small. We observed numerous significant differences between the genomic landscape of g*BRCA1* and g*BRCA2* PV–associated breast cancers, with implications for future therapeutic selection. When evaluating ER-positive, HER2-negative treatment-naive breast cancers, we found significant enrichment of genetic changes associated with CDK4/6i resistance mechanisms.

Primary analysis of POSH found no difference in breast cancer OS in g*BRCA1/2* PV carriers compared with noncarriers. Other studies also have found similar breast cancer OS comparing g*BRCA1/2* PV carriers and noncarriers following treatment with anthracyclines and taxane chemotherapy, although improved pathological complete response rates to chemotherapy in g*BRCA1* PV carriers are seen (59, 60). When *BRCA1/2* asLOH status was considered, breast cancer OS for nonLOH tumors was better than but not significantly different from OS for asLOH tumors; OS for asLOH and nonLOH tumors was significantly better than OS for non*BRCA1/2* tumors (14). In ovarian cancer, asLOH tumors had significantly better OS than nonLOH tumors and non-*BRCA1/2* tumors after platinum treatment (14). Our findings are suggestive of better outcomes for women with nonLOH tumors but are limited by their low sample count and are not statistically significant. We also observed significantly improved OS in tumors without SBS3. It is possible that tumors with asLOH and SBS3 have genomic instability that promotes progression despite initial responses to chemotherapy. Larger studies are needed to clarify whether nonLOH tumors indeed have more favorable outcomes.

Comparing g*BRCA1* and g*BRCA2* breast cancers with asLOH, g*BRCA1* tumors had significantly higher TMB scores (although the median TMB was low at 3.4), HRD scores, and proportions of aging-associated SBS1 and ROS-associated SBS18. g*BRCA2* asLOH tumors had higher proportions of SBS3 and SBS26, which are associated with defective DNA repair through homologous recombination and mismatch repair, respectively (37, 61). g*BRCA1* tumors, compared with g*BRCA2* tumors, had enrichment of multiple chromosome arm gains and amplifications; chr6q was the most significant even after correction for ER status. Other studies also have found greater numbers of CNVs in g*BRCA1* than in g*BRCA2* tumors, focal gains and amplifications of chr6q in g*BRCA1* tumors, and deletions of chr6q in g*BRCA2* tumors (22, 23, 62, 63). We show that the deletions of chr6q that are significantly enriched in g*BRCA2* tumors are dependent on ER status, as the association is lost with adjustment for ER status, and that chr6q gains are g*BRCA1* specific. Chr6q contains the cancer-associated genes *ROS1* (64); *FOXO3*, which, in addition to promoting breast cancer growth and metastasis, regulates ROS (65, 66); and *RSPO3*, which drives the formation of hormone receptor–negative breast cancer and is associated with epithelial-mesenchymal transition (EMT) (67). We find significantly more copy number gains of *ROS1*, *FOXO3*, and *RSPO3* and significantly more *FOXO3* amplifications in g*BRCA1* compared with g*BRCA2* tumors. Compared with g*BRCA2* tumors, g*BRCA1* tumors were enriched for alterations in ROS, EMT, and DNA repair pathways. Loss of *BRCA1* has been shown to promote EMT, increase ROS, and dysregulate DNA repair pathways in other studies (24, 68–70). The observed molecular differences suggest that g*BRCA1* and g*BRCA2* tumors may respond differently to molecularly targeted therapies such as ROS1 inhibitors (64, 71) and anti-RSPO3 antibodies (72, 73), which are under investigation for cancer treatment in the preclinical and clinical settings.

It has been suggested that *RB1* asLOH occurs simultaneously with *BRCA2* asLOH in tumors with g*BRCA2* PVs owing to the proximity of the genes on chr13 (17, 54, 74). Inagaki-Kawata et al. suggested that *TP53* loss on chr17 co-occurred with *BRCA1* loss but did not determine whether the loss occurred on the same or different segments (17). We found that *RB1* asLOH was significantly more frequent in g*BRCA2* tumors than in g*BRCA1* tumors and co-occurred on CNV segments with *BRCA2* asLOH. The rate of *RB1* asLOH was greater than 70% in both g*BRCA1* and g*BRCA2* treatment-naive tumors. This finding suggests that *RB1* loss in the context of HRD may confer an intrinsic fitness benefit that is not necessarily *BRCA2* specific; however, loss of *RB1* likely happens through differing mechanisms in g*BRCA1* versus g*BRCA2* tumors, the latter being due to simultaneous loss of *BRCA2* and *Rb1* as they are in close physical proximity. There was no difference in the frequency of *TP53* asLOH between g*BRCA1* and g*BRCA2* tumors; the frequency of *TP53* asLOH was greater than 90% in both tumor types, and *TP53* asLOH was rarely on the same segments as *BRCA1* asLOH. A previous study suggested that during g*BRCA1*-associated breast cancer development, *TP53* asLOH occurred before *BRCA1* loss (75). Thus, *RB1* asLOH is dependent on *BRCA2* asLOH but *TP53* asLOH is independent of *BRCA1* asLOH in breast cancer.

The availability of FDA-approved targeted therapies for the treatment of g*BRCA1/2*-driven tumors and for ER-positive, HER2-negative tumors presents a treatment decision in the management of g*BRCA1/2* carriers with ER-positive, HER2-negative tumors (27, 28, 31–34, 76). Previous studies of breast tumors unselected for age at diagnosis, with ages ranging from 26 to 80 years, showed levels of asLOH as low as 54% in g*BRCA2* PV carriers (14). In this study of young women, we found high levels of asLOH, greater than 90%, in both g*BRCA1* and g*BRCA2* PV carriers, regardless of tumor ER status. Additionally, women with nonLOH breast cancers had improved survival, although to a non-significant degree, likely owing to small numbers. Similarly, women whose breast cancers did not demonstrate SBS3 consistently had improved survival but non-significantly when stratified by gene or ER status. This finding suggests that most breast cancers in young women with g*BRCA1/2* PVs may be responsive to PARP inhibition. It is likely that in populations with older g*BRCA1/2* carriers, sporadic tumors are mixed in with g*BRCA1/2*-associated breast cancers, but young patients have mostly g*BRCA1/2*-associated tumors. Two retrospective studies in patients with advanced breast cancer have suggested that g*BRCA2* PV carriers with advanced breast cancer have attenuated responses to CDK4/6i plus endocrine therapy (77, 78). Bruno et al. reported lower progression-free and overall survival in carriers of g*BRCA1/2*, g*ATM*, and g*CHEK2* PVs compared with noncarriers, and Kim et al. identified g*BRCA2* PVs as being associated with lower progression-free survival following treatment with CDK4/6i, albeit with only 5 g*BRCA2* PV carriers (77, 78). A larger study in patients with metastatic breast cancer similarly reported lower progression-free survival rates for g*BRCA2* PV carriers treated with CDK4/6i compared with noncarriers (54). Our findings in young women with early breast cancer suggest that genomic mechanisms that underlie resistance to CDK4/6 inhibitors, including SNVs/indels and CNVs in *RB1*, *TP53*, *FAT1*, and *MYC*, are already present in treatment-naive breast cancers. These findings support the need for prospective clinical trials to test the optimal choice between PARPi and CDK4/6i, such as the ongoing clinical trial (NCT06380751) testing PARPi versus CDK4/6i in g*BRCA1/2* PV carriers with metastatic breast cancer. Additionally, this study provides information that may be useful for clinicians making treatment decisions before prospective trials are completed (76).

The main limitation was the low number of participants with nonLOH tumors, which may have limited our ability to detect significant survival differences. We evaluated only pretreatment samples and thus can only make predictions about potential treatment responses that will need to be tested in subsequent studies.

In conclusion, we show that tumors from young g*BRCA1/2* PV carriers with breast cancer almost always have asLOH. Women with tumors without LOH or SBS are likely to have improved survival at baseline. We also show differences between the molecular features of g*BRCA1* and g*BRCA2* tumors that may impact the suitability of molecularly targeted therapies. Importantly, we show enrichment of gene alterations associated with resistance to CDK4/6 inhibition in ER-positive, HER2-negative tumors from g*BRCA1/2* PV carriers compared with noncarriers in treatment-naive breast cancers. Given the high levels of asLOH and presence of CDK4/6i resistance–associated alterations, our data suggest that PARPi may be preferable over CDK4/6i in young g*BRCA1/2* carriers with ER-positive, HER2-negative breast cancer when both therapies are considered in the adjuvant and metastatic setting, supporting the need for prospective trials testing these drugs.

## Methods

### Sex as a biological variable.

All participants in the study were female, reflecting the higher breast cancer risk that female g*BRCA1/2* PV carriers have compared with males.

### Study description.

The POSH prospective cohort study (United Kingdom, 2000–2008) recruited over 3,000 women with invasive breast cancer, age ≤40, to investigate the impact of g*BRCA1/2* PVs on breast cancer outcomes (10). The primary outcome analysis was published in 2018 with a median follow-up of 8.2 years (3).

Participants with g*BRCA1/2* PVs were identified using sequencing and multiplex ligation probe analysis in patients meeting UK thresholds for clinical germline genetic testing at the time (3, 79). Three hundred ninety-one patients with a g*BRCA1/2* PV were identified; 55 patients were excluded, as they received neoadjuvant chemotherapy. An additional 135 patients were excluded because tumor blocks were unavailable.

### Sequencing and bioinformatics analysis.

All H&E-stained sections were reviewed to ensure breast cancer diagnosis by a board-certified pathologist. After exclusion of patient samples with insufficient tumor as judged by the pathologist, failed DNA extraction, or failed library preparation, and those without tumor-germline matches, we performed WES of 160 formalin-fixed, paraffin-embedded (FFPE) tumor blocks and 194 germline DNAs (Figure 1). After tumor-germline matching, removal of duplicate tumor samples, and exclusion of 3 tumors that were metastatic at diagnosis, 2 tumors from patients without g*BRCA1/2* PVs, and 1 tumor sample with poor sequencing coverage, we had 136 matched tumor-germline sequenced samples (Figure 1). The only significant differences between the 136 and the full set of 391 g*BRCA1/2* PV carriers from POSH were receipt of neoadjuvant chemotherapy, pathological M stage, and unknown pathological T stage, all of which were due to predetermined study eligibility criteria (Supplemental Table 1). The eligibility for the overall study included women diagnosed with breast cancer between ages 41 and 50 years if known to be g*BRCA1/2* positive. We called and filtered somatic single-nucleotide variants (SNVs), indels, and copy number variants (CNVs) in 598 cancer genes (Supplemental Table 2) using a custom computational pipeline (Supplemental Figure 1).

We selected 81 women with treatment-naive, non-metastatic, ER-positive, HER2-negative invasive breast cancer diagnosed before age 50 years without g*BRCA1/2* PVs (14) in TCGA for use as noncarrier comparators. We retrieved BAM files from the National Cancer Institute Genomic Data Commons (80), excluding 6 patients with germline PVs in *CHEK2*, *LZTR1*, *MSH6*, *PALB2*, and *POT1*. Somatic SNVs, indels, and CNVs were called using the same custom computational pipeline used for POSH samples in 66 unique tumor-germline samples (Supplemental Figures 1 and 8).

Details of asLOH determination, HRD score calculation, single-base substitution (SBS) signature fitting, and TMB calculation are given in Supplemental Methods.

### Statistics.

We performed Firth logistic regression to determine the enrichment of chromosomal arm CNVs and alterations in genes by germline variant gene, ER status, and HRD status (threshold HRD-high ≥42) in R (81). Analyses were adjusted for ER status, germline variant gene, asLOH status, and multiple testing as appropriate. We compared mean HRD scores using 2-sided *t* tests and compared median SBS proportions and TMB scores using Mann-Whitney and Kruskal-Wallis non-parametric tests. Survival analysis was performed using the Kaplan-Meier survival estimator; we estimated hazard ratios (HRs), 95% confidence intervals (CIs), and *P* values using Cox regression with Firth’s penalized method.

We assigned genes to pathways based on hallmark pathway (50) classification and performed enrichment analysis using Firth logistic regression, as above, with the hallmark pathways as dependent variables. Analyses were adjusted for ER status, germline variant gene, and asLOH status as appropriate.

*P* < 0.05 was considered statistically significant.

### Study approval.

Written informed consent was obtained from all participants at recruitment for further analysis of their tissue and clinical data; ethical approval was granted in 2000 by the South West Multicentre Research Ethics Committee (MREC00/6/69).

### Data availability.

All data necessary for the analyses performed in this study are provided in the Supplemental Tables and the Supporting Data Values file. Tumor sequencing data are available in the NCBI’s Sequence Read Archive database (PRJNA1374212). Analytic code is available at https://github.com/nathanson-lab/POSH_WES_JCI_Insight_2026, commit ID d83a320. Anonymous germline sequencing data from the POSH study will be available upon request starting 3 months after publication of the article, for the purpose of an approved proposal, to researchers who have provided a completed Data Sharing request form that describes a methodologically sound proposal and, if appropriate, signed a Data Sharing Agreement. Proposals will be reviewed by the POSH study committee. Data will be shared once all parties have signed relevant data sharing documentation, covering the study steering committee conditions for sharing, and, if required, an additional Data Sharing Agreement from the sponsor. Proposals for POSH study data should be directed to the chief investigator of the POSH study at D.M.Eccles@soton.ac.uk.

## Author contributions

HM, WT, ERC, RIC, and DME collected, provided, and transferred the samples. AN evaluated and annotated tumors on FFPE blocks. MPA, KPD, MF, JSS, and DP extracted DNA and prepared sequencing libraries. BW and MX performed bioinformatics analysis of the sequencing data. MPA and MX analyzed the data. WT, ERC, RIC, SMD, DME, and KLN directed analysis and obtained funding. MPA, MX, and WC wrote the manuscript. All authors critically reviewed the manuscript. KLN supervised the work.

## Conflict of interest

ERC has received honoraria from AstraZeneca, Eli Lilly, Guardant, Menarini Stemline, Novartis, Pfizer, and Roche; has served on advisory boards or provided consultation for AstraZeneca, Eli Lilly, NanoString, Pfizer, and Roche; has received conference fees or payment for travel/accommodation from Roche and Novartis; and has received an educational grant from Daiichi Sankyo. ERC and RIC have received research support from Seca.

## Funding support

- Breast Cancer Research Foundation (KLN and SMD).
- Basser Center for BRCA (KLN and SMD).
- Gray Foundation (KLN).
- Funding for the POSH study provided by the Wessex Cancer Trust (N.133; to DME), Cancer Research UK (C1275/A7572, C1275/A11699, C1275/A19187; to DME), Breast Cancer Now (2005NOV53; to DME), and Prevent Breast Cancer (GA23-05; to WT).
- The long-term follow-up of the POSH study is supported by an Institutional Grant from AstraZeneca (70053237; to ERC).

## Supplementary Material

Supplemental data

ICMJE disclosure forms

Supplemental tables 1-10

Supporting data values

## Figures and Tables

**Table 1:**
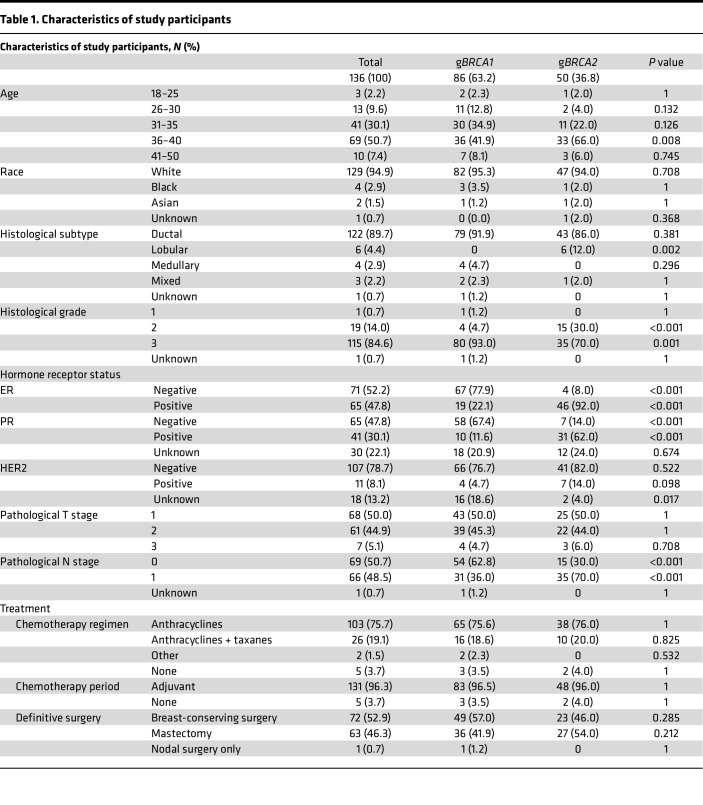
Characteristics of study participants

## Version 1 04/28/2026

In-Press Preview

## Version 2 06/22/2026

Electronic publication
