# Supplementary material for: Comparative analysis of distinct genomic landscapes in young-onset gBRCA1/2 breast cancer
Source: JCI Insight. 2026 Apr 28;11(12):e203005. doi: 10.1172/jci.insight.203005 (PMC13313557; doi:10.1172/jci.insight.203005)
Supplement: Supplemental data [file jciinsight-11-203005-s147.pdf]

Supplemental Material

Supplemental Methods

Whole-exome sequencing (WES) of tumor and germline DNA

Germline DNA libraries were prepared using the NEBNext Ultra DNA Library Prep Kit (New England Biolabs). Tumor DNA was extracted from FFPE using standard laboratory deparaffinization, proteinase K digestion, and ethanol precipitation. Tumor DNA libraries were prepared using the NEBNext FFPE DNA Repair Mix and NEBNext Ultra II DNA Library Prep Kit (New England Biolabs). DNA libraries were pooled and hybridized using SureSelect Human All Exon v7 for Illumina Multiplexed Sequencing (Agilent). All DNA samples, libraries, and hybridization pools were quantified with Qubit 2.0 Fluorometer (ThermoFisher), and fragment size was determined by BioAnalyzer 2100 (Agilent). WES was performed with 150 paired end reads on Illumina NovaSeq 6000 by the University of Pennsylvania Next Generation Sequencing Core.

Bioinformatics Analysis: Data Preprocessing

Tumor and germline BAM files were aligned to the GRCh38 reference genome using Burrows-Wheeler Aligner (BWA v.0.7.17) (1). Sequencing quality metrics were generated by Samtools (v.1.20) (2). Pathogenic germline variants in *BRCA1/2* were called by DeepVariant (v.1.6.0) and VarDict and were confirmed with prior clinical sequencing records for each patient at the University of Southampton (3, 4). We implemented an “identity by descent” (IBD) algorithm (PLINK v.1.9), using common biallelic SNV (SNP) locations to confirm matching of tumor and germline DNA samples with IBD scores > 0.9 (5, 6).

Bioinformatics Analysis: Identification of Somatic Pathogenic SNV and Indels

Somatic single-nucleotide variants (SNV) and indels were called and filtered using a custom computational pipeline (Supplemental Figure 1). SNVs and indels were first called by a combination of Lancet (v.1.1.0), Mutect2 (v.4.2.0.0), Strelka2 (v.2.9.2), VarDict, and Varscan2 (v.2.4.4), and then annotated using Ensembl Variant Effect Predictor (VEP v.112) (4, 7-11). All somatic variants were preprocessed using Varlociraptor (v.8.4.6) and custom scripts to remove potential sequencing and FFPE artifacts (12). We selected variants with Varlociraptor somatic variant probability ≥ 0.9 in cancer genes (Supplemental Table 2) for inclusion in our analysis for POSH but not TCGA samples because the lower sequencing depth in the TCGA samples skewed the Varlociraptor probability calls (13-15). We cleaned variants to remove those we determined as duplicates, such as SNVs called by Strelka2 and Varscan, which overlapped with multi-nucleotide variants called by Lancet/Mutect2/Vardict, and indels within a 50bp window of a single gene. We excluded upstream and downstream gene variants from the analysis. We also excluded insertions and indels >5bp in length that were called by Vardict only because the caller does not output BAMs, preventing visual validation of the calls in Integrative Genomics Viewer (IGV). Smaller insertions and indels could be reviewed using BAM files from the alignment by BWA.

From the clean set of variants, we selected those that were annotated as pathogenic, likely-pathogenic, or pathogenic/likely pathogenic in ClinVar (16). We used AutoGVP (17) to resolve variants with conflicting interpretations of pathogenicity. We also selected protein-coding variants annotated as oncogenic or likely oncogenic by OncoKB. Additionally, we developed custom criteria to profile variants without ClinVar or OncoKB

records (Supplemental Figure 1). We used ANNOVAR (18) (November 2023 release) to annotate our data with gnomAD v.4.1.0 (19) allele frequencies and selected variants with allele frequencies ≤ 0.5%, excluding variants annotated benign or likely-benign in ClinVar, or neutral or likely neutral in OncoKB. Of these variants, we selected stop-gained, frameshift, splicing (SpliceAI delta score ≥ 0.5), and start-lost variants, accounting for escape of nonsense-mediated decay for those within tumor suppressor genes (TSG) (as assigned in COSMIC and OncoKB) (20, 21).

<u>Bioinformatics Analysis: Calculation of tumor mutational burden (TMB) and single-base substitution signatures (SBS)</u>

Tumor mutational burden (TMB) was calculated according to the following formula: $\frac{(N\ somatic,\ exonic\ nonsynonymous\ SNV\ \&\ MNV + indels)}{size\ \ of\ WES\ capture\ (Mbp)}$ and single-base substitution (SBS) signatures (COSMIC) and their contributions to each tumor sample were identified using deconstructSigs (22, 23). Tobacco smoking/chewing signatures, unknown signatures, and possible sequencing artefacts, as described by COSMIC, were excluded from the contribution calculation (22). Only variants with alternative allele depth > 20, alternative allele frequency > 0.01, Varlociraptor (12) somatic variant probability ≥ 0.9, and allele frequency ≤ 0.005 in gnomAD v.4.1.0 were used for determining TMB and SBS.

<u>Bioinformatic Analysis: Identification of somatic pathogenic CNV, determination of allele-specific loss of heterozygosity, and calculation of homologous recombination deficiency</u>

Allele-specific copy number variants (CNV) were called by a combination of ASCAT (v.3.1.3), FACETS (v.0.6.2), CNVkit (v.0.9.11)/PureCN (v.2.4.0) with segmentation by CNVkit and allele-specific calls by PureCN, and Sequenza (v.3.0), and were annotated

by AnnotSV (v.3.4.2) (24-29). We filtered CNV by cancer genes and sorted by TSG and oncogenes as assigned in COSMIC and OncoKB. We called CNV variants in the TCGA samples using only FACETS and CNVkit.

In TSG, we selected biallelic loss, as defined by copy number 0 in both alleles, noted by at least 2 callers. In the oncogenes, we selected gain (total copy number = 3 or 4) and amplification (total copy number ≥ 5) with a consensus from at least 3 callers. When consensus was not reached, we calculated the average copy number and designated calls as 1) biallelic loss if average copy number was <0.5, 2) gain if average CN was >2.5 and <4.5, and 3) amplification if average total copy number was ≥4.5.

To evaluate the CNV landscape by chromosomal arm, we obtained genomic coordinates for each arm from the UCSC genome browser. The average total copy number from ASCAT, FACETS, PureCN, and Sequenza was calculated for each arm. We designated the calling for each arm based on the following rules: 1) loss if average total copy number was <1.5, 2) gain if average total CN was ≥2.5. The visualization is representative of the ASCAT segmental calls in IGV.

To evaluate joint loss of *RB1-BRCA2* and *TP53-BRCA1*, annotated allele-specific copy number variation (CNV) segments (including genomic positions and allele-specific copy numbers) generated by ASCAT were compiled for *BRCA1*, *BRCA2*, *RB1* and *TP53* in each tumor sample. For all tumors with allele-specific loss of heterozygosity in *RB1* or *TP53*, we noted the genomic coordinates of the beginning and end of the segment with copy loss that overlapped with each gene. We then determined how many times each lost segment overlapped with *RB1* only or *RB1* and *BRCA2*, or with *TP53* only, or *TP53*

and *BRCA1*. We performed Chi-square tests to determine whether joint loss occurred more frequently than expected by chance.

Allele-specific LOH at *BRCA1/2* was noted if the tumor contained 1) a second somatic PV in the gene with the germline PV; or 2) allele-specific copy loss of the wildtype allele called by at least two out of four CNV callers used: ASCAT, FACETS, PureCN, and Sequenza. CNVs were annotated using AnnotSV (24-28).

Genomic scars HRD score, defined as the sum of genomic LOH, telomeric allelic imbalance, and large state transitions, was calculated using HRDex with LOH values averaged from ASCAT, PureCN and Sequenza used as input (30).

Supplemental references

1. Li H, and Durbin R. Fast and accurate short read alignment with Burrows–Wheeler transform. *Bioinformatics.* 2009/07/15;25(14):1754-60.

2. Danecek P, Bonfield JK, Liddle J, Marshall J, Ohan V, Pollard MO, et al. Twelve years of SAMtools and BCFtools. *GigaScience.* 2021/01/29;10(2).

3. Poplin R, Chang P-C, Alexander D, Schwartz S, Colthurst T, Ku A, et al. A universal SNP and small-indel variant caller using deep neural networks. *Nature Biotechnology 2018 36:10.* 2018-09-24;36(10):983-7.

4. Lai Z, Markovets A, Ahdesmaki M, Chapman B, Hofmann O, McEwen R, et al. VarDict: a novel and versatile variant caller for next-generation sequencing in cancer research. *Nucleic Acids Research.* 2016/06/20;44(11):e108.

5. Anderson CA, Pettersson FH, Clarke GM, Cardon LR, Morris AP, and Zondervan KT. Data quality control in genetic case-control association studies. *Nature Protocols.* 2010;5(9):1564-73.

6. Purcell S, Neale B, Todd-Brown K, Thomas L, Ferreira MAR, Bender D, et al. PLINK: A Tool Set for Whole-Genome Association and Population-Based Linkage Analyses. *The American Journal of Human Genetics.* 2007/09/01;81(3):559-75.

7. Narzisi G, Corvelo A, Arora K, Bergmann EA, Shah M, Musunuri R, et al. Genome-wide somatic variant calling using localized colored de Bruijn graphs. *Communications Biology 2018 1:1.* 2018-03-22;1(1):20.

8. Benjamin D, Sato T, Cibulskis K, Getz G, Stewart C, and Lichtenstein L. Calling Somatic SNVs and Indels with Mutect2. *bioRxiv.* 2019-12-02.

9. Kim S, Scheffler K, Halpern AL, Bekritsky MA, Noh E, Källberg M, et al. Strelka2: fast and accurate calling of germline and somatic variants. *Nature Methods 2018 15:8.* 2018-07-16;15(8):591-4.

10. Koboldt DC, Zhang Q, Larson DE, Shen D, McLellan MD, Lin L, et al. VarScan 2: Somatic mutation and copy number alteration discovery in cancer by exome sequencing. *Genome Research.* 2012-03-01;22(3):568-76.

11. McLaren W, Gil L, Hunt SE, Riat HS, Ritchie GRS, Thormann A, et al. The Ensembl Variant Effect Predictor. *Genome Biology 2016 17:1.* 2016-06-06;17(1):122.

12. Köster J, Dijkstra LJ, Marschall T, Schönhuth A, Köster J, Dijkstra LJ, et al. Varlociraptor: enhancing sensitivity and controlling false discovery rate in somatic indel discovery. *Genome Biology 2020 21:1.* 2020-04-28;21(1):98.

13. Sondka Z, Dhir NB, Carvalho-Silva D, Jupe S, Madhumita M, McLaren K, et al. COSMIC: a curated database of somatic variants and clinical data for cancer. *Nucleic Acids Research.* 2024/01/05;52(D1):D1210-D7.

14. Chakravarty D, Gao J, Phillips S, Kundra R, Zhang H, Wang J, et al. OncoKB: A Precision Oncology Knowledge Base. *JCO Precision Oncology.* 2017-05-16;1(1).

15. Suehnholz SP, Nissan MH, Zhang H, Kundra R, Nandakumar S, Lu C, et al. Quantifying the Expanding Landscape of Clinical Actionability for Patients with Cancer. *Cancer Discovery.* 2024/01/01;14(1):49-65.

16. Landrum MJ, Lee JM, Riley GR, Jang W, Rubinstein WS, Church DM, et al. ClinVar: public archive of relationships among sequence variation and human phenotype. *Nucleic Acids Research.* 2014/01/01;42(D1):D980-5.

17. Kim J, Naqvi AS, Corbett RJ, Kaufman RS, Vaksman Z, Brown MA, et al. AutoGVP: a dockerized workflow integrating ClinVar and InterVar germline sequence variant classification. *Bioinformatics.* 2024;40(3):btae114.

18. Wang K, Li M, and Hakonarson H. ANNOVAR: functional annotation of genetic variants from high-throughput sequencing data. *Nucleic Acids Research.* 2010/09/01;38(16):e164.

19. Karczewski KJ, Francioli LC, Tiao G, Cummings BB, Alföldi J, Wang Q, et al. The mutational constraint spectrum quantified from variation in 141,456 humans. *Nature 2020 581:7809.* 2020-05-27;581(7809):434-43.

20. Jaganathan K, Kyriazopoulou Panagiotopoulou S, McRae JF, Darbandi SF, Knowles D, Li YI, et al. Predicting Splicing from Primary Sequence with Deep Learning. *Cell.* 2019;176(3):535-48.e24.

21. Lindeboom RGH, Supek F, Lehner B, Lindeboom RGH, Supek F, and Lehner B. The rules and impact of nonsense-mediated mRNA decay in human cancers. *Nature Genetics 2016 48:10.* 2016-09-12;48(10):1112-8.

22. Alexandrov LB, Kim J, Haradhvala NJ, Huang MN, Tian Ng AW, Wu Y, et al. The repertoire of mutational signatures in human cancer. *Nature.* 2020;578(7793):94-101.

23. Rosenthal R, McGranahan N, Herrero J, Taylor BS, Swanton C, Rosenthal R, et al. deconstructSigs: delineating mutational processes in single tumors distinguishes DNA repair deficiencies and patterns of carcinoma evolution. *Genome Biology 2016 17:1.* 2016-02-22;17(1):31.

24. Ross EM, Haase K, Van Loo P, and Markowetz F. Allele-specific multi-sample copy number segmentation in ASCAT. *Bioinformatics.* 2021/07/27;37(13):1909-11.

25. Shen R, and Seshan VE. FACETS: allele-specific copy number and clonal heterogeneity analysis tool for high-throughput DNA sequencing. *Nucleic Acids Research.* 2016/09/19;44(16):e131.

26. Riester M, Singh AP, Brannon AR, Yu K, Campbell CD, Chiang DY, et al. PureCN: copy number calling and SNV classification using targeted short read sequencing. *Source Code for Biology and Medicine 2016 11:1.* 2016-12-15;11(1):13.

27. Favero F, Joshi T, Marquard AM, Birkbak NJ, Krzystanek M, Li Q, et al. Sequenza: allele-specific copy number and mutation profiles from tumor sequencing data. *Annals of Oncology.* 2015/01/01;26(1):64-70.

28. Geoffroy V, Herenger Y, Kress A, Stoetzel C, Piton A, Dollfus H, et al. AnnotSV: an integrated tool for structural variations annotation. *Bioinformatics.* 2018/10/15;34(20):3572-4.

29. Talevich E, Shain AH, Botton T, and Bastian BC. CNVkit: Genome-Wide Copy Number Detection and Visualization from Targeted DNA Sequencing. *PLoS Comput Biol.* 2016;12(4):e1004873.

30. Pluta J, Hausler R, Wubbenhorst B, Desai H, Domchek SM, Nathanson KL, et al. HRDex: a tool for deriving homologous recombination deficiency (HRD) scores from whole exome sequencing data. *bioRxiv.* 2022-09-12.

Supplemental Tables (attached excel spreadsheet)

Supplemental Table 1: Characteristics of *BRCA1/2* pathogenic variant carriers in the full POSH study and in the current study

Supplemental Table 2: List of cancer genes evaluated

Supplemental Table 3: Homologous recombination deficiency scores and tumor mutational burden in germline *BRCA1* and *BRCA2* pathogenic variant carriers

Supplemental Table 4: Single base substitution signatures in germline *BRCA1* and *BRCA2* pathogenic variant carriers Supplemental Table 5: Clinical characteristics of tumors without loss of heterozygosity (nonLOH)

Supplemental Table 6: Frequency of variants identified in tumors

Supplemental Table 7: Significantly altered genes by gene variant

Supplemental Table 8: Significantly altered genes by estrogen receptor status

Supplemental Table 9: Significantly altered genes in homologous recombination deficiency high and low tumors

Supplemental Table 10: Significantly altered genes in tumors with and without allele-specific loss of heterozygosity

Supplemental Figures

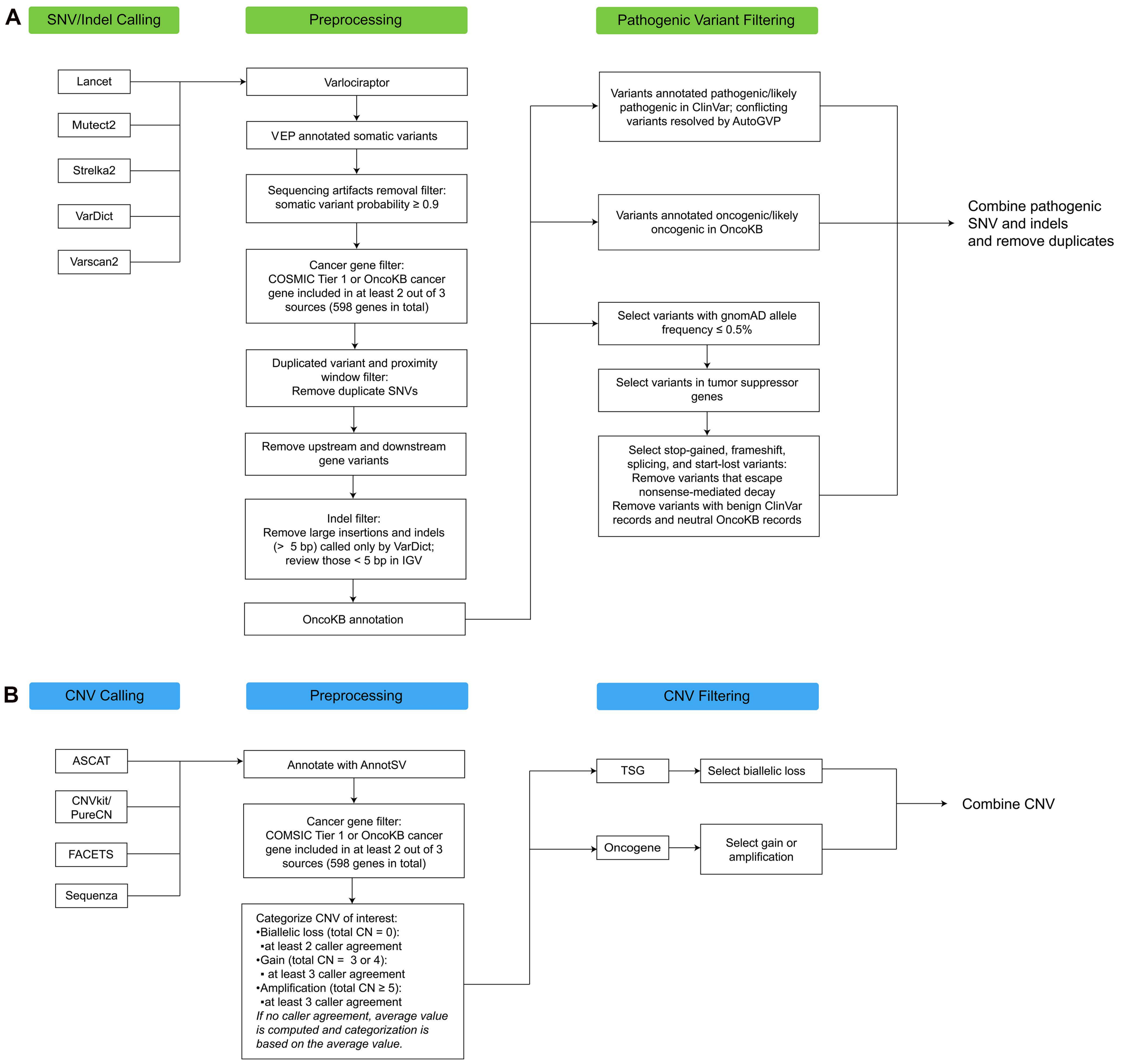

Supplemental Figure 1: Custom somatic variant analysis pipeline.

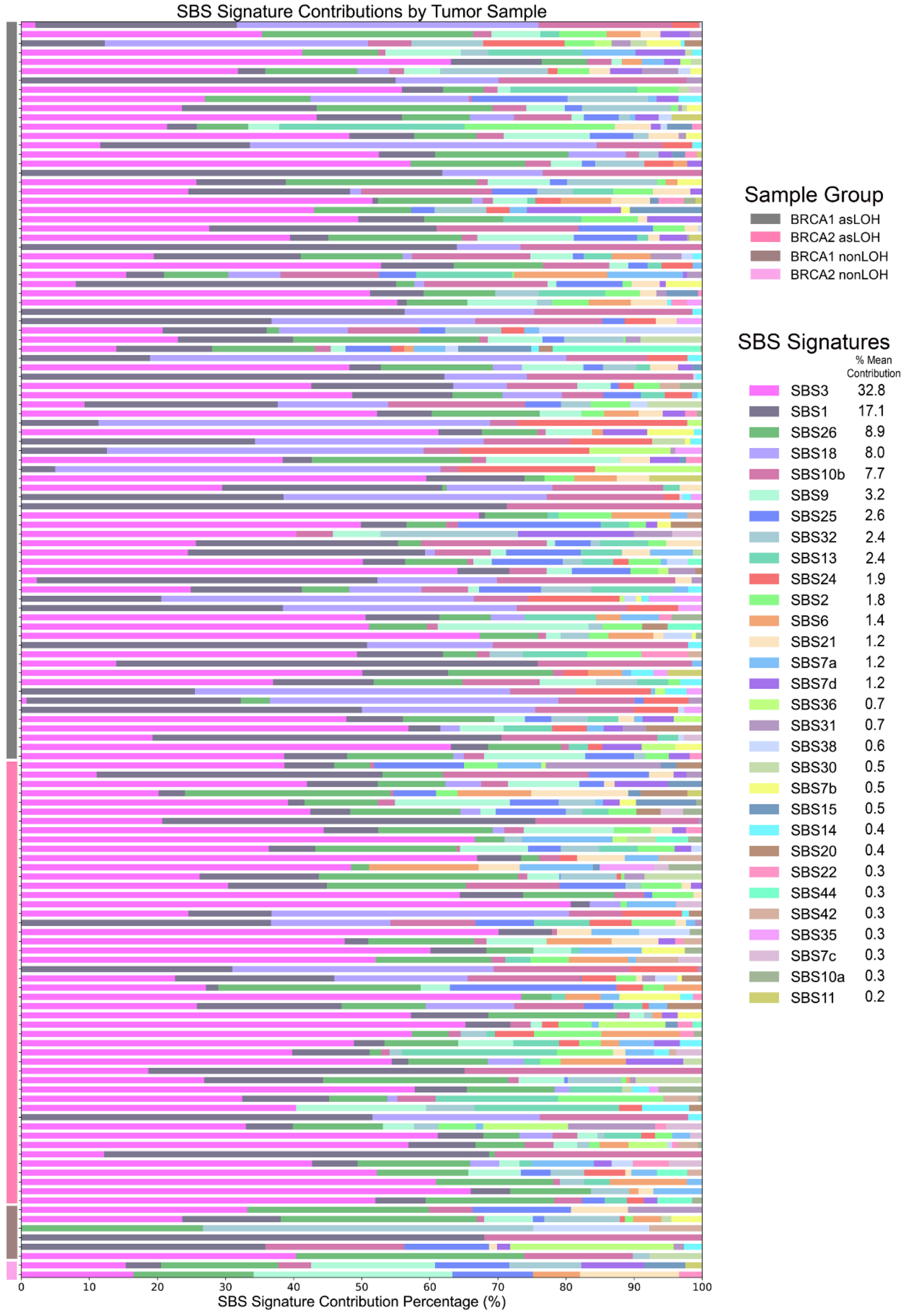

Supplemental Figure 2: Landscape of single base substitutions in tumors in POSH.

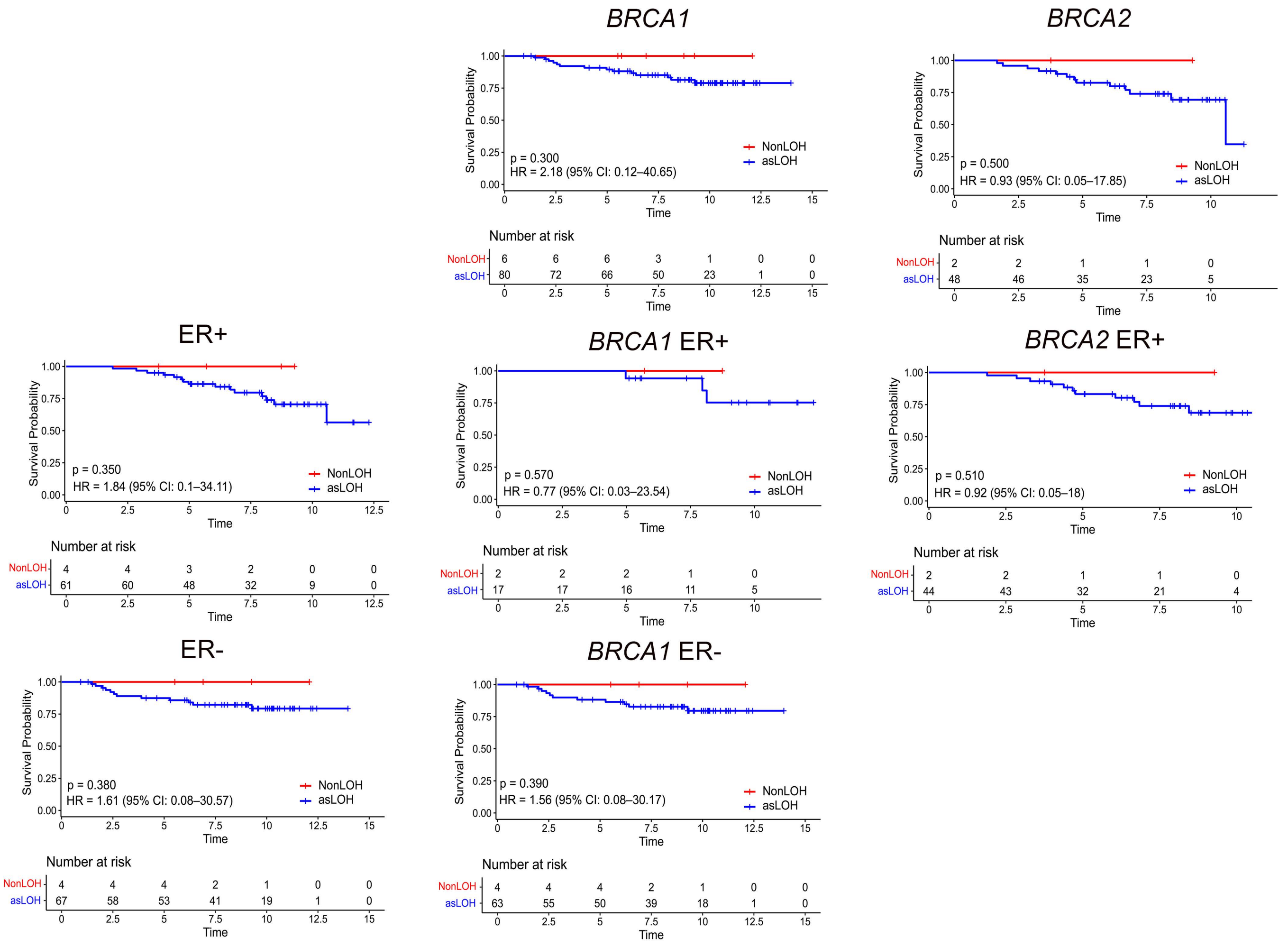

Supplemental Figure 3: Overall survival by loss of heterozygosity status.

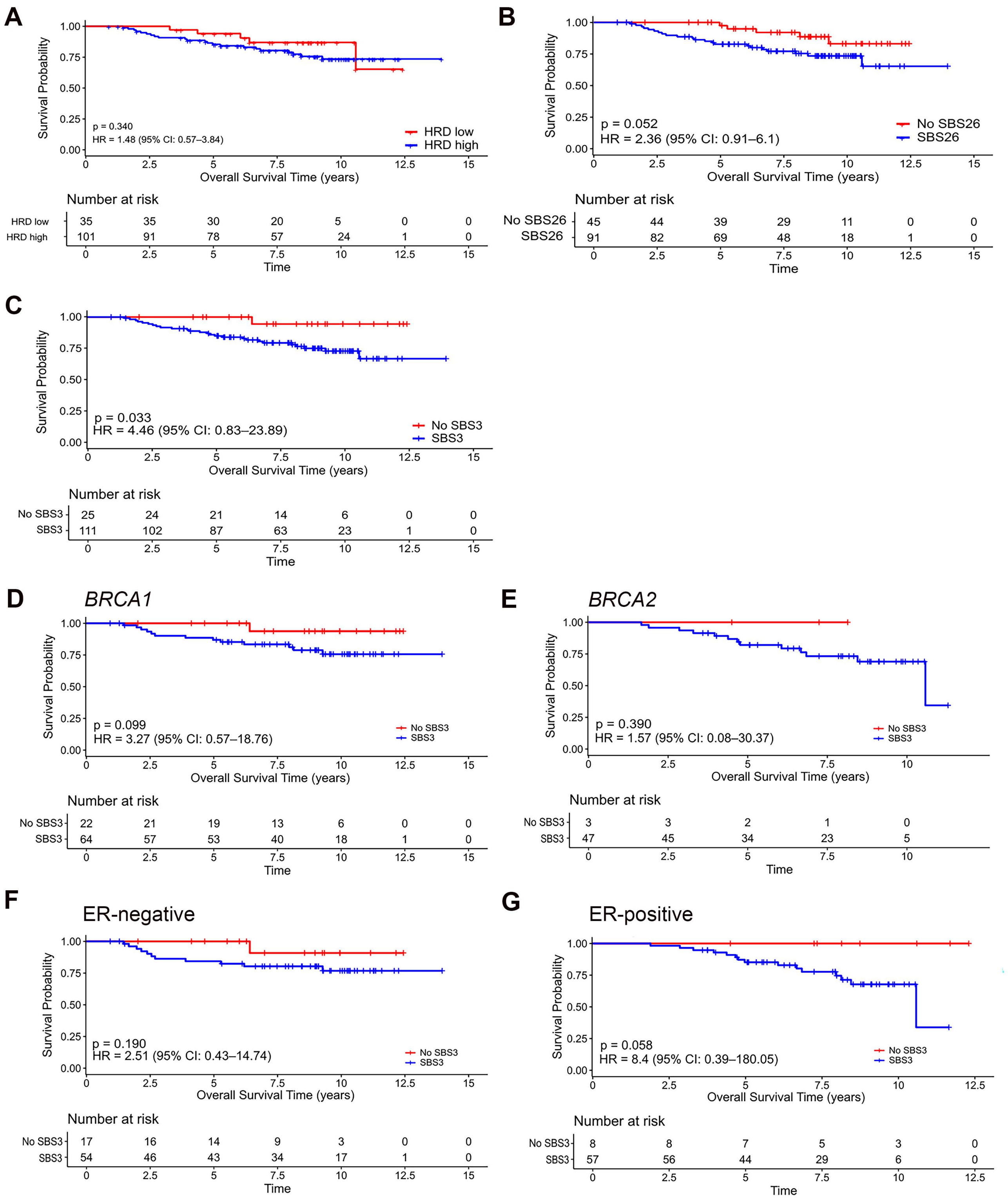

Supplemental Figure 4: Overall survival by A) homologous recombination deficiency (HRD), B) single base substitution signature 26 (SBS26), C) overall SBS3, and SBS3 in D) *BRCA1*-associated tumors, E) *BRCA2*-associated tumors, F) estrogen receptor (ER)-negative, and G) ER-positive tumors. HR = hazard ratio.

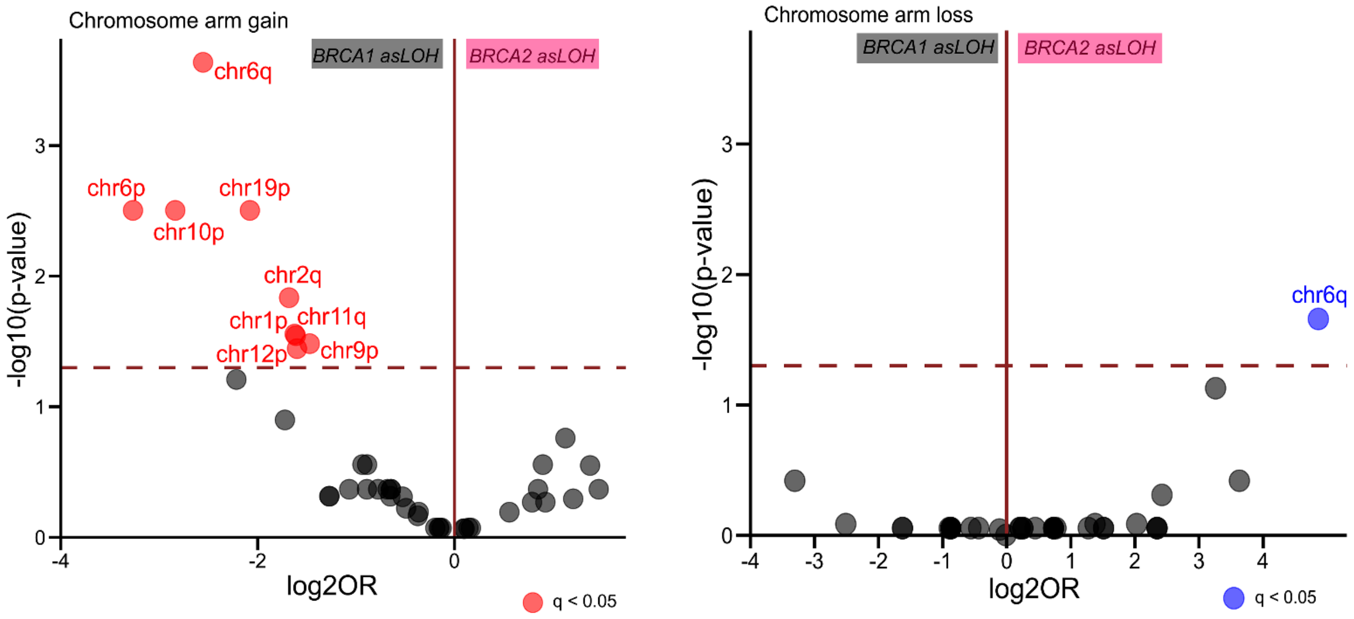

Supplemental Figure 5: Differential enrichment of chromosome arm copy changes in *BRCA1* and *BRCA2* tumors with asLOH adjusted for multiple testing.

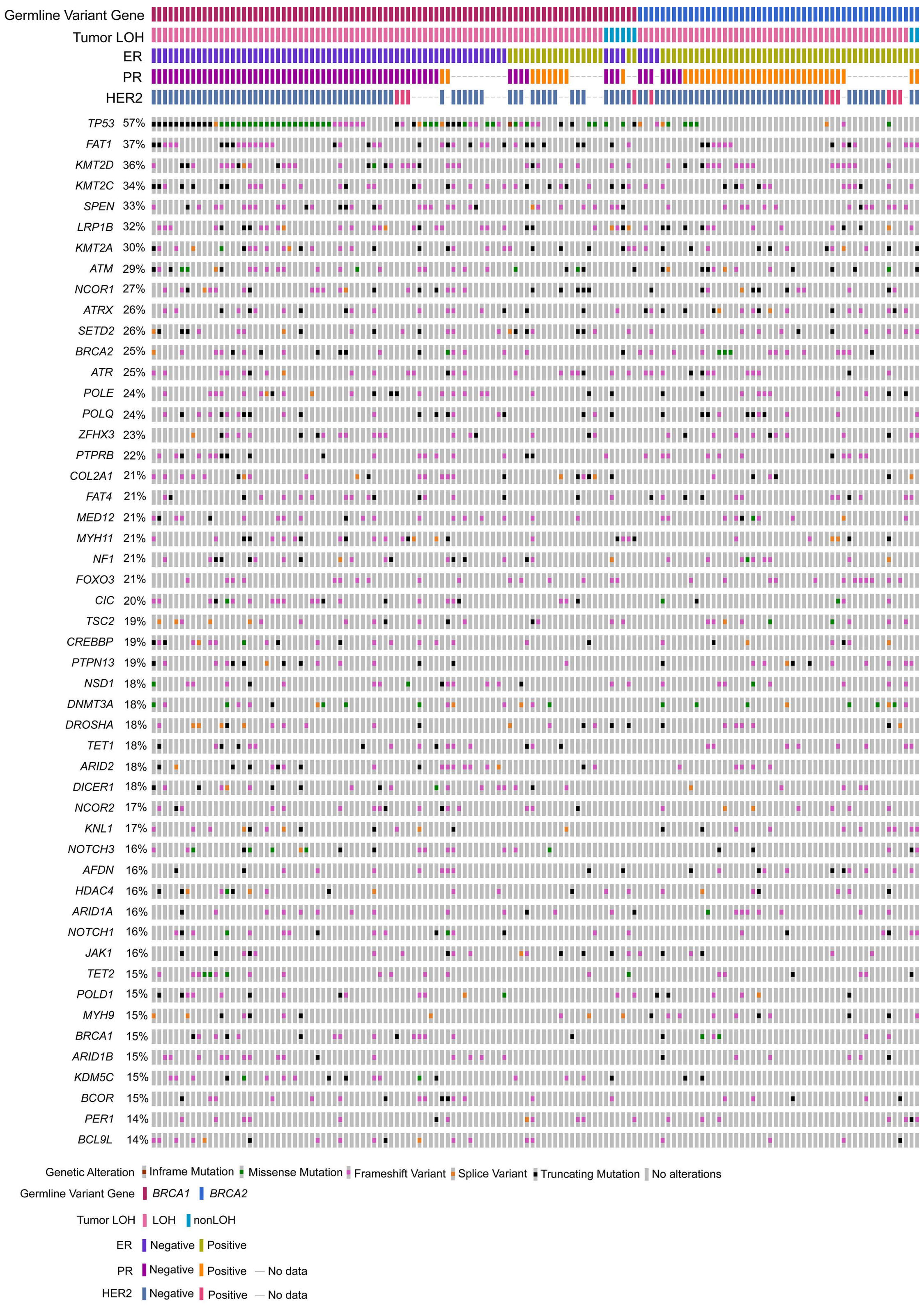

Supplemental Figure 6: Oncoprint of the 50 most altered genes by single nucleotide variants and indels.

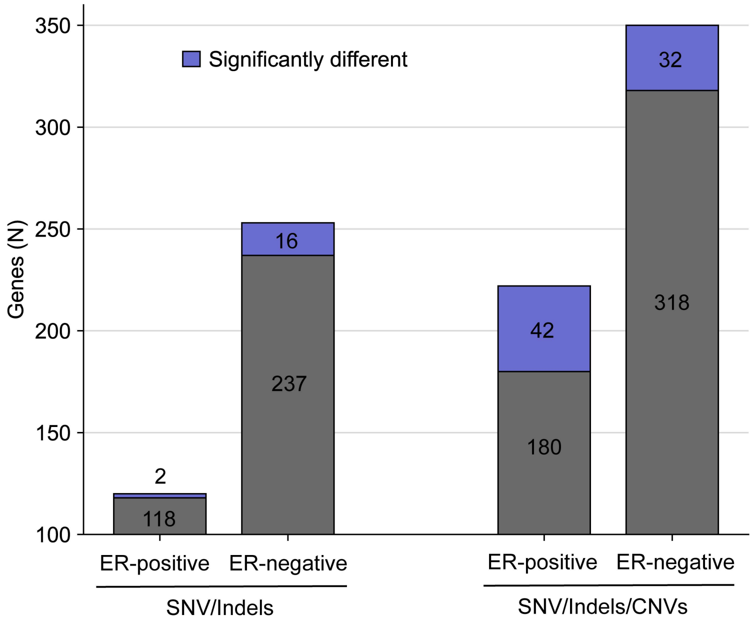

Supplemental Figure 7: Number of genes with single nucleotide variants/indels/copy number variants by estrogen-receptor status.

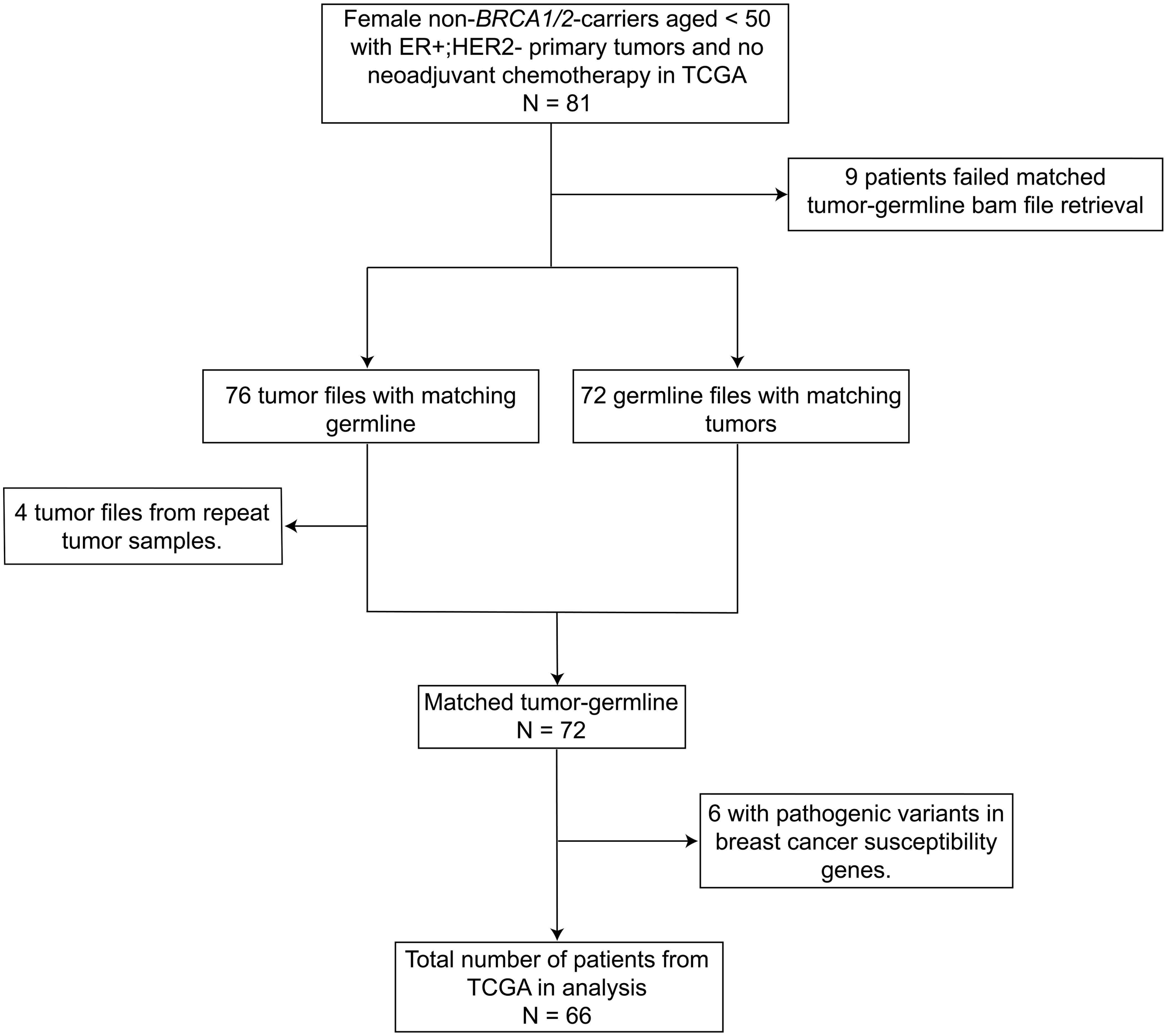

Supplemental Figure 8: CONSORT diagram of TCGA samples used in this study.
